# RNase 7 in Cutaneous Defense

**DOI:** 10.3390/ijms17040560

**Published:** 2016-04-14

**Authors:** Franziska Rademacher, Maren Simanski, Jürgen Harder

**Affiliations:** Department of Dermatology, University of Kiel, 24105 Kiel, Germany; frademacher@dermatology.uni-kiel.de (F.R.); msimanski@dermatology.uni-kiel.de (M.S.)

**Keywords:** RNase 7, cutaneous defense, antimicrobial peptides (AMPs)

## Abstract

RNase 7 belongs to the RNase A superfamily and exhibits a broad spectrum of antimicrobial activity against various microorganisms. RNase 7 is expressed in human skin, and expression in keratinocytes can be induced by cytokines and microbes. These properties suggest that RNase 7 participates in innate cutaneous defense. In this review, we provide an overview about the role of RNase 7 in cutaneous defense with focus on the molecular mechanism of the antimicrobial activity of RNase 7, the regulation of RNase 7 expression, and the role of RNase 7 in skin diseases.

## 1. Introduction

Natural RNase 7 was first isolated from *stratum corneum* extracts due to its antimicrobial activity against several pathogenic microorganisms [1]. RNase 7 exhibits also ribonuclease activity and is a member of the RNase A superfamily [2]. Members of this family are characterized by its unique three-dimensional disulfide-bonded structure and are homolog to the bovine pancreatic ribonuclease A (RNase A) [3]. On genomic level 13, human members have been identified on chromosome 14 [4]. RNases 1–8 are typical members of the canonical RNase A family containing the catalytic site necessary for ribonuclease activity. RNases 9–13 have the characteristic disulfide-bonded structure but are unlikely to exhibit ribonuclease activity. Several members of the human canonical RNase A family members share antimicrobial features. For example, the eosinophil-derived human RNase 2 (EDN) and RNase 3 (ECP) exhibit antiviral activities [5,6]. In addition, there is increasing evidence that RNase 3, RNase 5 (angiogenin), RNase 7, and RNase 8 may play an important role in host defense due to their antimicrobial activities [7,8,9,10].

**Figure 1:**
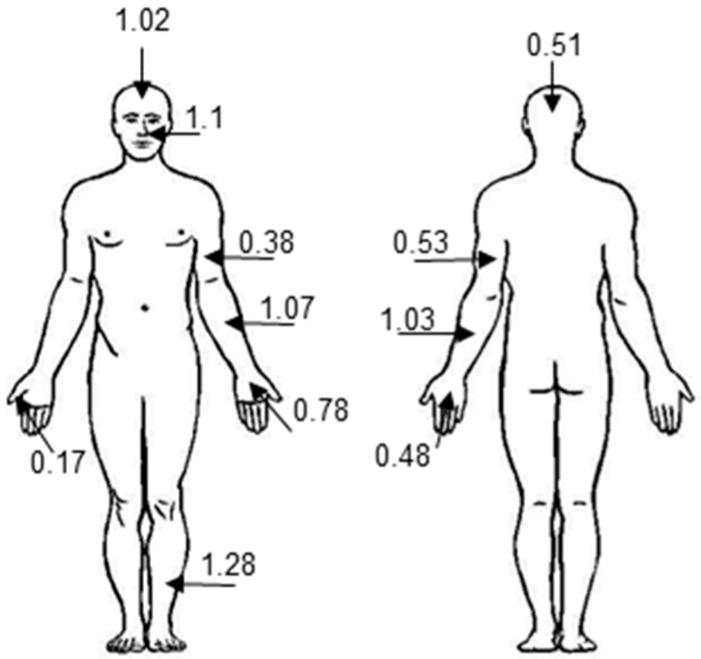
RNase 7 concentration is site-dependent. Defined skin areas of probands (*n* = 10) were rinsed with 10 mM of sodium phosphate buffer containing 150 mM of NaCl, pH 7.4. The RNase 7 protein concentration was determined by ELISA. The depicted RNase 7 concentrations refer to ng/cm^2^ [13]. Data are mean values (*n* = 10).

*RNASE7* mRNA is expressed in various epithelial tissues with highest amounts in the skin, tonsils, pharynx, and the genito-urinary tract [1,11,12]. Further analyses identified keratinocytes as expression source of RNase 7 in human skin. An analysis of RNase 7 secreted on the skin surface at various body sites revealed a localization-dependent pattern (Figure 1).

Additionally to its constitutive expression in keratinocytes RNase 7 is inducible by proinflammatory cytokines (e.g., interferon-γ, interleukin-1β, interleukin-17A, and -17C) as well as by microbial stimuli like bacteria (e.g., *Pseudomonas* *aeruginosa*, *Staphylococcus* *aureus*, *E. coli*, and *Enterococcus* *faecium*) [1,13,14]. The abundance of RNase 7 in skin and its inducibility by cytokines and microorganisms together with its antimicrobial activity indicates that RNase 7 participates in cutaneous defense.

Interestingly, there is no structural RNase 7 orthologue in mouse making *in vivo* studies difficult [7]. It has been suggested that the common ancestor of the *RNASE7* gene and its closely related *RNASE8* gene emerged from the duplication of the *RNASE6* gene prior to the primate-rodent divergence and that lower vertebrates including mouse do not harbor the *RNASE7* gene [4]. Perhaps specific environmental characteristics and microbial threats exerted an evolutionary pressure on the presence of RNase 7 in higher organisms. Here, we summarize the current state of knowledge about the role of RNase 7 in cutaneous defense.

## 2. Antimicrobial Activity of RNase 7

### 2.1. Microbial Targets

RNase 7 exhibits high antimicrobial activity against a broad spectrum of microorganisms. Among the *in vitro* tested microorganisms are Gram-positive and Gram-negative bacteria like *Pseudomonas* *aeruginosa*, *Staphylococcus* *aureus*, *Enterococcus* *faecium*, the yeast *Candida* *albicans* and *Pichia* *pastoris*, and the dermatophyte *Trichophyton* *rubrum* [1,13,15,16].

Using antibodies that block the antimicrobial activity of RNase 7, we investigated the contribution of RNase 7 to the cutaneous antibacterial chemical defense shield. We could show that natural RNase 7 was highly effective in killing the Gram-positive bacterium *Enterococcus* *faecium*, an abundant member of the gut flora but also an opportunistic pathogen. Moreover, *stratum corneum* displayed a potent bactericidal activity towards *Enterococcus* *faecium*, and this activity could be significantly reduced by inactivation of RNase 7 by a blocking antibody [13]. These results indicate that RNase 7 contributes to the capacity of the *stratum corneum* to control the growth of *Enterococcus* *faecium*. The production of antimicrobial peptides and proteins (AMPs) such as RNase 7 may explain why the skin at the perianal region is highly resistant to infection caused by Enterococci despite the permanent contact with these abundant gut bacteria.

*Staphylococcus* *aureus* (*S. aureus*) is an important Gram-positive pathogenic bacterium that causes various skin infections. Using skin explants, we have shown that skin infected with living *S.* *aureus* responds with an increased release of RNase 7. Application of a specific RNase 7 blocking antibody led to an increased growth of *S. aureus* on the skin surface. These findings highlight the important role of RNase 7 in cutaneous defense to limit the growth of *S. aureus* [14].

Zanger *et al.* [17] studied the association between the level of constitutive RNase 7 expression in healthy skin and the tendency to develop *S. aureus* infection. Therefore, they compared 20 travelers returning with *S. aureus*—a positive skin infection—with a control group and documented that RNase 7 expression in unaffected skin of the control group was 64% higher than in unaffected skin of the infected individuals. This was not the case for the AMPs human β-defensin (hBD)-2 and hBD-3. The authors calculated an approximate doubling in risk of infection for a 30% lower level of *RNASE7* gene expression [17]. This was the first report linking altered RNase 7 expression in healthy skin with susceptibility to cutaneous *S. aureus* infection. It highlights the importance of RNase 7 to protect human skin from bacterial infection.

RNase 7 is also abundantly expressed in the urinary tract. Therefore, Spencer and colleagues analyzed the relevance of RNase 7 to protect the urinary tract from infection with uropathogenic bacteria [11]. They incubated urine samples from healthy donors with either uropathogenic *E. coli*, *Pseudomonas*, *Enterococcus*, *Klebsiella*, or *Proteus* *mirabilis*. Addition of a blocking RNase 7 antibody led to increased bacteria growth. These data suggest that RNase 7 plays an important role in the innate defense of the human uroepithelium.

Pulido *et al.* investigated the antimicrobial potential and mechanisms of RNase 7 and RNase 3 against *Mycobacterium* *vaccae* [18]. Both RNases inhibited the mycobacterial growth *in vitro* suggesting that RNase 3 and RNase 7 may also have a role in host defense against mycobacteria.

### 2.2. Mechanism

The mechanism of the antimicrobial activity of RNase 7 is not fully understood. Because RNase 7 is a ribonuclease the question arises whether the RNA degrading activity is important for its antimicrobial activity. Huang *et al.* used RNase 7 mutants (H15A, K38A, and H123A) lacking RNase activity to show that the enzymatic RNA degrading activity is not essential for the bactericidal activity of RNase 7 against *Pseudomonas* *aeruginosa* [16]. In line with these results, we found that recombinant mutated RNase 7 without ribonuclease activity retained its capacity to kill *Enterococcus* *faecium* [13].

Furthermore, Huang *et al.* describe three clusters of cationic residues on the surface of RNase 7 tertiary structure. Substitution of cationic residues in the first N-terminal cluster led to reduced antimicrobial activity. In contrast, the other two cationic clusters including the catalytic residues were not critical for bactericidal activity [16]. These results indicate that lysine residues K1, K3, K111, and K112 in the first cationic cluster are pivotal for the bactericidal activity of RNase 7.

Recent functional studies suggest that the antimicrobial properties of RNase 7 are retained in its N-terminal domain, a feature which is common for all antimicrobial active members of the human RNase A superfamily [19].

Binding experiments and the results of a SYTOX green cell viability assay lead to the assumption that RNase 7 binds to the negatively charged surface of the bacterial membrane and disrupts it via electrostatic interactions [16]. Using artificial liposomes, Torrent and colleagues reported that RNase 7 binds to the membrane and causes local membrane destabilization [20]. The authors concluded that interaction with the membrane must be electrostatically driven because RNase 7 did not interact with uncharged liposomes. Studies with bacteria gave more insight in the antimicrobial activity. It has been reported that RNase 7 is able to depolarize the cytoplasmatic membrane of *E. coli* and *S. aureus* and to bind to lipopolysaccharide (LPS) and peptidoglycan, main components of the bacterial cell wall of Gram-negative and Gram-positive bacteria, respectively [21]. The bactericidal activity of RNase 7 occurred by leakage, and no bacterial aggregation was observed as reported for RNase 3 [21].

Lin *et al.* have reported that the outer membrane lipoprotein OprI (outer membrane protein I) of *P. aeruginosa* serves as the initial binding site for RNase 7. The importance of OprI was documented by addition of exogenous OprI or anti-OprI antibody, resulting in diminished antimicrobial activity of RNase 7. Based on their data, the authors hypothesize that binding of RNase 7 to OprI leads to processing of OprI and internalization of OprI into the bacterial cytosol together with RNase 7 [22]. This study indicates that RNase 7 targets specific surface-associated bacterial lipoproteins. Further studies are needed to show whether other bacteria express similar surface-associated proteins targeted by RNase 7.

The ribonuclease activity of the members of the RNase A superfamily including RNase 7 can be inhibited by the endogenous ribonuclease inhibitor (RI). The RI has the form of a horseshoe and binds RNases of the RNase A superfamily in a ratio of 1:1 [23,24,25]. This raises the question of whether binding of the RI to RNase 7 may also affect antimicrobial activity. In human keratinocytes, the mRNA and protein concentration of RNase 7 and the RI was increased during keratinocyte differentiation [26]. When the RI was added to RNase 7 *in vitro*, the ribonucleolytic as well as the antimicrobial activity of RNase 7 were strongly reduced. Whereas immunohistochemical analyses located RNase 7 mainly in the *stratum corneum* the RI was nearly absent in the *stratum corneum*. This could be explained by the presence of proteolytic activity of the *stratum corneum*, which led to *in vitro* degradation of the RI but not RNase 7 [26]. These data suggest that the RI may regulate the ribonucleolytic and the antimicrobial activity of RNase 7 in the human epidermis [26].

Spencer and colleagues described that RI expression is higher in the differentiated superficial umbrella cells than in the basal stem cells of the uroepithelium, similarly to the expression pattern in the skin [27]. The RI mRNA and protein levels decreased with acute pyelonephritis and the investigators detected full length and degraded RI in infected urine. This RI degradation may be catalyzed by neutrophil proteases (proteinase 2 and elastase) [27]. Alternatively, the authors speculate that the strong binding of RI to RNase 7 may be disabled due to effects of oxidative stress (e.g., reactive oxygen species (ROS) or reactive nitrogen intermediates). It is known that RI is very sensitive to oxidation due to the amount of reduced cysteine residues needed for RI activity. Together, the RI may also play an important role at the epithelial-urinary interface to control activity of RNase 7 [27].

### 2.3. Bacterial Strategies to Subvert the Antimicrobial Activity of RNase 7

As mentioned above, the cationic net charge of AMPs is important for their antimicrobial activity due to electrostatic interaction with the negatively charged surface of bacteria. Using a *S. aureus* strain deficient in d-alanylated teichoic acids (dltA mutant), we demonstrated that d-alanylation of its surface reduces the susceptibility of *S. aureus* to skin-derived AMPs such as RNase 7 and human β-defensins [28]. This observation was accompanied by a higher killing activity of skin extracts towards the *S. aureus* dltA mutant as well as towards clinical isolates expressing lower levels of dltA. We conclude that modulation of the cell envelope d-alanylation may help *S. aureus* to persist on human skin through evasion of cutaneous innate defense provided by cationic skin-derived AMPs such as RNase 7 [28].

*S. aureus* is able to change its phenotype into small-colony variants (SCVs). This phenotype grows slower and takes longer to become visible on agar plates, which may often lead to undetected bacteria followed by incorrect diagnostics [29,30]. Gläser *et al.* investigated whether this phenotype influences the susceptibility to the antimicrobial activity of skin-derived AMPs [31]. Therefore, they analyzed natural SCV clinical isolates and their corresponding isogenic wild-type strains. The SCV showed a decreased susceptibility to the tested AMPs RNase 7, hBD-2, hBD-3, and LL-37 as compared to the corresponding wild-type strains. The molecular mechanism explaining why SCVs show a higher resistance to AMPs is not clear. One reason may be the lower electrochemical gradient across the bacterial cell membrane [32]. The authors conclude that switching into the SCV phenotype may help *S. aureus* to subvert cutaneous innate defense, thus contributing to the establishment and persistence of infection [31].

## 3. Regulation of RNase 7 Expression

RNase 7 was originally isolated from human *stratum corneum* extracts by Harder and colleagues during a search for AMPs that are present in healthy skin [1]. Analysis of *RNASE7* gene expression in different tissues confirmed high expression of *RNASE7* in the skin. Besides skin, *RNASE7* gene expression was also detected in various other tissues, indicating that skin is not the only source of *RNASE7* gene expression and that RNase 7 may also play important roles in other epithelial tissues [1]. For example, as mentioned above Spencer and colleagues observed high expression of RNase 7 in the urinary tract and they demonstrated an important role of RNase 7 to protect the urinary tract from infection [11,33].

In human skin immunostaining of RNase 7 revealed the highest expression in the *stratum corneum* [13]. This observation was also shown by immunohistochemistry analysis of fetal human skin from the second trimester of pregnancy [34]. Immunostaining of RNase 7 expression in biopsies from nasal mucosa revealed the highest RNase 7 expression in the luminal cell layers of the *stratum corneum* from the *vestibulum nasi* [35]. Intensive staining of RNase 7 in the *stratum corneum* was also detected in an immunohistochemistry analysis of atopic dermatitis and psoriasis skin [36]. The finding that RNase 7 expression is mainly located in the outermost layers of human skin correlates with the supposed function of RNase 7 to protect human skin from infection.

The abundant constitutive expression of RNase 7 in human keratinocytes might be an important factor for the prevention of skin infections. Moreover, RNase 7 expression is inducible by various proinflammatory cytokines like interleukin1-β (IL-1β), interleukin-17A (IL-17A), and interferon-γ (IFNγ) in nasal epithelial cells [37], human corneal epithelial cells [38], and keratinocytes [39]. IL-1β was identified as a potent inductor for RNase 7 expression in human corneal epithelial cells by Mohammed and colleagues. They concluded that IL-1β-induced RNase 7 expression in human corneal epithelial cells is mediated by activation of the mitogen-activated protein kinase (MAPK) pathway [38]. The inducibility of RNase 7 expression in keratinocytes and nasal epithelial cells by proinflammatory cytokines was significantly higher by using cytokine combinations [37,39]. Burgey and colleagues identified IFNγ in comparison with TNFα and IL-1β as the most potent cytokine inducing RNase 7 expression in nasal epithelial cells. A 29-fold higher induction of RNase 7 expression after 24 h was achieved by using a cytokine combination of IFNγ, IL-1β, and TNFα [37].

Simanski and colleagues identified the combination of IL-17A/IFNγ as the most potent tested cytokine mix to induce RNase 7 expression in keratinocytes. IL-17A/IFNγ-induced RNase 7 expression was decreased by a specific STAT3 inhibitor as well as by STAT3-siRNA [39]. STAT3 mutations have been identified to cause the inflammatory disease Hyper IgE-syndrome (HIES). HIES patients often suffer from epithelial infections such as cutaneous infection caused by *S. aureus* [40]. Thus, one may speculate that STAT3-mediated induction of AMPs such as RNase 7 may be compromised in patients with HIES, thereby contributing to the increased susceptibility to cutaneous infection. 

A characteristic of RNase 7 expression is its inducibility by bacteria such as *S. aureus* [14,41], *P. aeruginosa* [1], *S. epidermidis* [41], and the dermatophyte *Trichophyton rubrum* [42]. Firat and colleagues reported on an induction of RNase 7 expression in keratinocytes by the dermatophyte *Trichophyton* *rubrum*. This *Trichophyton* *rubrum*-mediated RNase 7 induction was synergistically enhanced by the cytokine combination IL-17A/IFNγ [42]. Other AMPs such as hBD-3 were also induced in keratinocytes infected with *Trichophyton* *rubrum*, indicating that keratinocytes are able to sense the presence of dermatophytes leading to an increased release of AMPs. Responsible pattern recognition receptors (PRRs) are not identified yet, but toll-like receptors (TLRs), which are upregulated in tinea [43] or C-type lectin-like receptors, such as dectin-1 and dectin-2, which are described as a binding component of *Trichophyton* *rubrum*, may be involved [44].

There is increasing evidence that the epidermal growth factor (EGFR) plays a key role in mediating RNase 7 expression in keratinocytes. The application of an EGFR inhibitor or an EGFR blocking antibody to *Trichophyton* *rubrum*-stimulated primary keratinocytes revealed a significant decrease of RNase 7 expression [42]. Lichtenberger and colleagues showed that blocking the EGFR pathway resulted in a decreased antimicrobial activity of cultured keratinocytes against *S. aureus*. This was accompanied by a reduced expression of RNase 7 in the keratinocytes treated with an EGFR inhibitor [45]. Wanke and colleagues demonstrated that the EGFR ligand transforming growth factor alpha (TGFα) induced gene expression of *RNASE7* in keratinocytes. In addition, they reported that the induction of *RNASE7* gene expression by culture supernatants of *S. epidermidis* was reduced in the presence of an EGFR inhibitor [41]. Together, these studies suggest that the EGFR signaling pathway may play a crucial role for RNase 7 expression and induction in keratinocytes. It is known that cancer patients who receive EGFR inhibition therapy are associated with an increased risk for cutaneous infections by diverse microorganisms including *S.* *aureus* and dermatophytes [46]. Thus, one may hypothesize that a reduced RNase 7 expression may contribute to the increased infection risk in patients receiving anti-EGFR therapy [45].

Wanke and colleagues reported that, in addition to an EGFR inhibitor, a TLR-2 blocking antibody as well as an inhibitor of the transcription factor NFκB decreased the induction of *RNASE7* gene expression in keratinocytes by secreted factors of *S. epidermidis*. In contrast, induction of *RNASE7* by secreted factors of *S. aureus* was not influenced by these inhibitors. Instead, secreted factors of *S. aureus* activated the mitogen-activated protein kinase and phosphatidylinositol 3-kinase/AKT signaling pathways [41]. Together, these results indicate that a pathogenic and a commensal member of the *Staphylococcus* genus induce *RNASE7* in keratinocytes via different signal transduction pathways.

**Figure 2:**
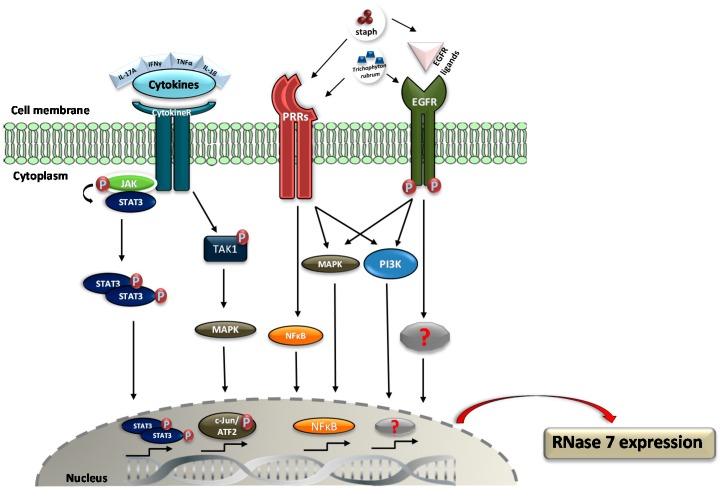
Regulation of RNase 7 induction. Mohammed *et al.* [38] reported that RNase 7 expression induced by IL-1β in human corneal epithelial cells is regulated by phosphorylation of transforming growth factor β-activated kinase-1 (TAK1) and subsequent activation of mitogen-activated protein kinase (MAPK) pathway leading to activation of the transcription factors c-Jun and ATF2. Simanski *et al.* [39] demonstrated that IL-17A/IFNγ-induced RNase 7 expression in keratinocytes is mediated via STAT3 signaling. Wanke *et al.* [41] reported that *Staphylococcus*-mediated *RNASE7* induction is regulated via two different pathways. *Staphylococcus* *epidermidis* activates TLR-2 and a signaling cascade involving activation of the EGFR by EGFR ligands as well as activation of the transcription factor NFκB. *Staphylococcus* *aureus* activates the mitogen-activated protein kinase and phosphatidylinositol 3-kinase (PI3K)/AKT signaling pathways. Firat *et al.* [14] documented that EGFR signaling is involved in *Trichophyton* *rubrum*-induced RNase 7 expression in keratinocytes. (PRRs = pattern recognition receptors; black arrows = activation; red curved arrow = induction; red “P” = phosphorylated).

In summary, there is clear evidence that the expression of RNase 7 in keratinocytes and other epithelial cells can be induced by microbial stimuli as well as by endogenous factors such as cytokines and growth factors. Figure 2 provides an overview about the underlying putative signaling transduction pathways leading to RNase 7 induction.

## 4. Role of RNase 7 in Skin Diseases

The high expression of RNase 7 in the skin together with its broad-spectrum antimicrobial activity makes it likely that RNase 7 may play a role in skin diseases. As mentioned above, several studies point to a relevance of RNase 7 in protecting healthy skin from infection. The question arises whether RNase 7 may also play a role during skin infection and inflammation. In the following section, we will summarize and discuss the current knowledge about the potential role of RNase 7 in skin diseases.

### 4.1. Atopic Dermatitis

*S.* *aureus* is the major pathogen isolated from infected skin of patients with atopic dermatitis (AD). Especially lesional AD skin is prone to superinfection caused by *S. aureus*. Several publications report on a reduced expression of AMPs in the lesional AD skin as compared to lesional psoriatic skin, which is normally free of infections. This led to the hypothesis that AD is associated with an insufficient induction of AMPs, leading to an increased susceptibility to *S. aureus* infection. However, whether a potentially decreased induction of AMPs predisposes AD skin to *S. aureus* infection is still under debate [47,48]. As discussed above, several reports ascribe RNase 7 an important role to control cutaneous growth of *S. aureus*. Thus, it is of interest to evaluate whether a dysregulated RNase 7 expression may be associated with AD and increased *S. aureus* growth. Surprisingly, a gene expression study with skin biopsies demonstrated not only a higher gene expression of *RNASE7* in lesions of AD as compared to healthy skin but detected also a significantly higher *RNASE7* gene expression in lesions of AD as compared to psoriatic lesions [49]. Similarly, analyses of RNase 7 levels secreted on the skin surface revealed significant higher RNase 7 concentrations on the lesional AD skin surface as compared to non-lesional AD skin or skin of healthy controls. Furthermore, levels of RNase 7 were similar on the skin surface of lesional skin of AD and psoriasis patients [36]. Immunostainings of RNase 7 in lesional AD skin as compared to healthy skin confirmed the increase of RNase 7 expression in the AD lesion [36,50]. No major differences of RNase 7 immunostainings in skin lesions of AD and psoriatic skin could be detected [36]. Taken together, these results suggest that a decreased cutaneous induction of RNase 7 is not associated with AD. However, one cannot exclude that a failure of RNase 7 to efficiently kill *S. aureus* may be associated with AD. Such inefficient antimicrobial activity could be caused by an inactivation of RNase 7 or by loss of antimicrobial potency due to structural changes based on SNPs or by an inadequate mobilization of RNase 7 onto surface-associated *S. aureus* as it has been reported for hBD-3 [51].

### 4.2. Psoriasis

As mentioned above, expression of RNase 7 is upregulated in the psoriatic lesion. This is also documented by high amounts of RNase 7 present in psoriatic scales [52]. In contrast to AD, psoriatic skin is characterized by a lower infection rate despite the disrupted barrier [53]. The high amounts of various AMPs such as β-defensins, RNases, S100-proteins, and cathelicidin present in the psoriatic lesion may—at least in part—offer an explanation for this unexpected high resistance. One may speculate that the increased amounts of RNase 7 contribute to the low infection rate seen in psoriasis.

The increased AMP levels detected in the psoriasis lesion may also trigger inflammation because various AMPs exhibit also immunomodulatory activities including proinflammatory activities such as induction of cytokine release and chemotactic activity towards immune cells [54]. For example, one of the highest induced factors in the psoriatic lesion is human β-defensin-2 (hBD-2). Since hBD-2 is able to chemo-attract and activate immune cells, one may speculate that increased hBD-2 expression trigger the inflammatory scenario in psoriasis [55,56,57]. Whether RNase 7 may also exhibit similar immunomodulatory activities that may play a role in psoriasis or other inflammatory skin diseases such as atopic dermatitis is not known. Other members of the human RNase A superfamily have been reported to exert such immunomodulatory activities. For example, human pancreatic ribonuclease (RNase 1) and eosinophil-derived neurotoxin (EDN, RNase 2) have been shown to induce dendritic cell maturation and activation [58]. Thus, it remains to be shown whether RNase 7 also exhibits similar immunomodulatory activities that may play a role in inflammatory diseases such as psoriasis or atopic dermatitis.

### 4.3. Acne Vulgaris

Acne vulgaris is a common chronic inflammatory disease of the pilosebaceous units. It is often associated with the presence of *Propionibacterium* *acnes*, which is thought to trigger inflammation in this disease [59]. Expression of RNase 7 in the outer root sheath of the hair follicle [60] and *in vitro* antimicrobial activity against *Propionibacterium* *acnes* [1] may indicate a role of RNase 7 to control growth of *Propionibacterium* *acnes in vivo*. Whether RNase 7 may play a role in acne remains to be shown.

### 4.4. Wounds

The role of RNase 7 in skin wounding is still emerging. Superficial skin injury led to a fast release (already after one hour) of RNase 7 on the skin surface. One may speculate that the rapid released RNase 7 was derived from preformed material [36]. In contrast, there is some experimental evidence that RNase 7 is not induced in deeper skin wounds. For example, a study using experimental sterile wounding of healthy gluteal skin reported even a downregulation of *RNASE7* gene expression upon wounding. *RNASE7* mRNA levels declined in the wounded skin to half the levels detected in healthy skin [61]. Similarly, the same group detected a 50% decline of *RNASE7* gene expression in skin lesions caused by group A streptococci as compared to healthy skin [62]. Another study reported that expression of RNase 7 in wounds of chronic venous ulcers was not induced [63]. Further studies are needed to show whether a potential failure to adequately induce RNase 7 may be functionally associated with a disturbed wound healing process. In this context, it remains to be shown whether RNase 7 may also influence cutaneous regeneration and differentiation processes.

### 4.5. Viral Infections

As mentioned above, some members of the RNase A superfamily exhibit antiviral activity, among them the RNase 2 (EDN) and RNase 3 (ECP) [20,21]. Since ribonuclease activity of these antiviral RNases seems to be crucial for full antiviral activity, it is tempting to speculate that the high ribonuclease activity of RNase 7 may also play a role in antiviral defense. However, to our knowledge, no direct antiviral activity of RNase 7 has been demonstrated so far. Whereas RNase 2 and RNase 3 have been shown to reduce the capacity of respiratory syncytial virus (RSV) to infect epithelia cells, RNase 7 did not show such activity against RSV [2]. Interestingly, keratinocytes infected with the dengue virus responded with an increased induction of *RNASE7* gene expression [64]. However, whether RNase 7 exhibits antiviral activity towards dengue virus has not yet been reported.

### 4.6. Skin Cancer

Scola *et al.* demonstrated a decreased expression of RNase 7 in actinic keratosis and further decreased RNase 7 levels in cutaneous squamous cell carcinoma as compared to healthy skin. They speculate that RNase 7 may act as a tumor suppressor, a hypothesis which remains to be proven by further studies [65].

## 5. Conclusions

Although the physiological role of RNase 7 is still emerging there is increasing evidence that RNase 7 plays an important role in cutaneous innate defense. It is likely that a dysregulation of RNase 7 may be associated with cutaneous inflammatory and infectious diseases. The application or targeted induction of RNase 7 may offer future therapeutical strategies.

## Abbreviations

AD	Atopic dermatitis
AMPs	Antimicrobial peptides and proteins
EGFR	Epidermal growth factor
ECP	Eosinophil cationic protein
EDN	Eosinophil-derived neurotoxin
IL	Interleukin
IFN	Interferon
hBD	Human beta-defensin
TLR	Toll-like receptor
SCV	Small-colony variant
